# In Vivo Study of Nasal Bone Reconstruction with Collagen, Elastin and Chitosan Membranes in Abstainer and Alcoholic Rats

**DOI:** 10.3390/polym14010188

**Published:** 2022-01-04

**Authors:** Fabricio Egidio Pandini, Fabíola Mayumi Miyauchi Kubo, Ana Maria de Guzzi Plepis, Virginia da Conceição Amaro Martins, Marcelo Rodrigues da Cunha, Vinicius Rodrigues Silva, Vinicius Barroso Hirota, Everton Lopes, Marcos Antonio Menezes, André Antonio Pelegrine, Tiago Negrão de Andrade, Amilton Iatecola, Bruna da Cruz Britto, Victor Augusto Ramos Fernandes, Luis Felipe Orsi Gameiro, Ronny Rodrigues Correia, Marcelo Lucchesi Teixeira, Getúlio Duarte Júnior, Carlos Henrique Bertoni Reis, Eliana de Souza Bastos Mazuqueli Pereira, Daniela Vieira Buchaim, Karina Torres Pomini, Daniel de Bortoli Teixeira, Rogerio Leone Buchaim, Edmir Américo Lourenço

**Affiliations:** 1 Department of Surgery (Otorhinolaryngology), Jundiaí Medical School, Jundiaí 13202-550, Brazil; fabriciopandini@hotmail.com (F.E.P.); edmir@g.fmj.br (E.A.L.); 2 Department of Implant Dentistry, Faculdade São Leopoldo Mandic, Campinas 13045-755, Brazil; fabismm@yahoo.com (F.M.M.K.); marcosmene@yahoo.com.br (M.A.M.); pelegrineandre@gmail.com (A.A.P.); 3 Interunit Postgraduate Program in Bioengineering (EESC/FMRP/IQSC), University of São Paulo (USP), São Carlos 13566-590, Brazil; amplepis@iqsc.usp.br (A.M.d.G.P.); cunhamr@hotmail.com (M.R.d.C.); 4 São Carlos Institute of Chemistry, University of São Paulo (USP), São Carlos 13566-590, Brazil; virginia@iqsc.usp.br; 5 Department of Morphology and Pathology, Jundiaí Medical School, Jundiaí 13202-550, Brazil; vinnyeduca@gmail.com (V.R.S.); tiagonandr@gmail.com (T.N.d.A.); amilton.iatecola@ceunsp.edu.br (A.I.); brunabritto@uol.com.br (B.d.C.B.); victorfernandes@g.fmj.br (V.A.R.F.); 6 Coordination of the Physical Education Course, University Center of the Americas (FAM), São Paulo 01304-001, Brazil; vbhirota@gmail.com; 7 Padre Anchieta University Center, Jundiaí 13210-795, Brazil; everton.lopes@anchieta.br; 8 Unimetrocamp University Center, Campinas 13035-500, Brazil; lfgameiro85@gmail.com; 9 Botucatu Medical School (FMB), São Paulo State University (UNESP—Univ Estadual Paulista), Botucatu 18618-687, Brazil; ronny.rodrigues@unesp.br; 10 Prosthodontics Department, Faculdade São Leopoldo Mandic, Campinas 13045-755, Brazil; marceloltx@gmail.com; 11 Unimar Beneficent Hospital (HBU), University of Marilia (UNIMAR), Marilia 17525-160, Brazil; drgetulioduarte@hotmail.com (G.D.J.); dr.carloshenriquereis@usp.br (C.H.B.R.); 12 Department of Biological Sciences, Bauru School of Dentistry (FOB/USP), University of São Paulo, Bauru 17012-901, Brazil; karinatp@usp.br; 13 Postgraduate Program in Structural and Functional Interactions in Rehabilitation, Postgraduate Department, University of Marilia (UNIMAR), Marília 17525-902, Brazil; elianabastos@unimar.br (E.d.S.B.M.P.); danibuchaim@alumni.usp.br (D.V.B.); daniel.dbt@hotmail.com (D.d.B.T.); 14 Teaching and Research Coordination of the Medical School, University Center of Adamantina (UniFAI), Adamantina 17800-000, Brazil; 15 Postgraduate Program in Animal Health, Production and Environment, University of Marilia (UNIMAR), Marília 17525-902, Brazil; 16 Graduate Program in Anatomy of Domestic and Wild Animals, Faculty of Veterinary Medicine and Animal Science, University of São Paulo (FMVZ/USP), São Paulo 05508-270, Brazil

**Keywords:** bone regeneration, alcohol intake, collagen, elastin, chitosan, scaffolds, bone repair

## Abstract

The aim of the present study was to evaluate the use of collagen, elastin, or chitosan biomaterial for bone reconstruction in rats submitted or not to experimental alcoholism. Wistar male rats were divided into eight groups, submitted to chronic alcohol ingestion (G5 to G8) or not (G1 to G4). Nasal bone defects were filled with clot in animals of G1 and G5 and with collagen, elastin, and chitosan grafts in G2/G6, G3/G7, and G4/G8, respectively. Six weeks after, all specimens underwent radiographic, tomographic, and microscopic evaluations. Bone mineral density was lower in the defect area in alcoholic animals compared to the abstainer animals. Bone neoformation was greater in the abstainer groups receiving the elastin membrane and in abstainer and alcoholic rats receiving the chitosan membrane (15.78 ± 1.19, 27.81 ± 0.91, 47.29 ± 0.97, 42.69 ± 1.52, 13.81 ± 1.60, 18.59 ± 1.37, 16.54 ± 0.89, and 37.06 ± 1.17 in G1 to G8, respectively). In conclusion, osteogenesis and bone density were more expressive after the application of the elastin matrix in abstainer animals and of the chitosan matrix in both abstainer and alcoholic animals. Chronic alcohol ingestion resulted in lower bone formation and greater formation of fibrous connective tissue.

## 1. Introduction

The consumption of alcoholic beverages, where ethanol is its main component, can act as a toxic element to several vital organs. Alcoholism is considered a chronic disease that causes a series of damages to the individual’s health, such as psychological, organic, and socioeconomic disorders, being a relevant public health problem, and generating high costs for health systems and society in general. In accidents, when facial fractures occur, approximately 41% of patients have nasal fractures and alcohol consumption was directly related to these traumas [1,2,3]. The nasal pyramid is a bony complex formed by the bones of the nose itself and joined superiorly by the frontal process and inferiorly by the maxillary bone. Owing to its prominent location in the face, fractures of the nasal bone are common among traumatic maxillofacial bone injuries [4,5].

Although alcoholics are at an increased risk of trauma and fractures, alcohol itself has a negative impact on bone metabolism. Ethanol can reduce the levels of osteocalcin, a marker of bone synthesis, and can affect the mechanisms of bone formation and/or degradation [6,7]. Molecular studies involving alcoholics have identified elevated levels of sclerostin, a protein found in areas of bone resorption and an inhibitor of the wingless (Wnt) signaling pathway, with a consequent reduction in the expression of the β-catenin protein. The Wnt/β-catenin signaling pathway is responsible for osteoblastogenesis, which, when compromised, affects the differentiation, function, and half-life of osteoprogenitor cells and interferes with the balance between bone neoformation and resorption [8,9,10].

Facial traumas frequently require surgical intervention. The technologies currently used in reconstructive surgery of the craniomaxillofacial region to treat bone loss or failure include biomaterials such as bone grafts, barrier membranes, bioactive factors, and cell therapies [11]. Membranes for guided bone regeneration (GBR) mimic the extracellular matrix, regulate the cell phenotype, and provide an appropriate microarchitecture for the infiltration, adhesion, and proliferation of osteogenic cells, i.e., they host cells that express and secrete pro-osteogenic factors involved in the promotion of bone healing and restoration of the underlying bone defect. This bioactive effect has also been demonstrated for cells and molecules intentionally incorporated into the membrane and/or implanted into the underlying defect, and may be combined with bone graft materials. Within this context, synthetic materials and natural derivatives have been used in clinical practice as membranes for GBR [12,13,14].

Materials composed of natural polymers such as collagen, elastin, and chitosan are becoming increasingly important as scaffolds for bone tissue engineering. Collagen is the main component of connective tissues and plays an important role in the structural support of tissue and in extracellular matrix-mediated communication. Collagen-containing natural membranes are the most common material used for GBR, with their presence or degradation not exerting any deleterious effect on tissues. However, their main limitation is the lack of stiffness [11,15,16]. In addition to low immunogenicity, collagen membranes provide support for angiogenesis, an important feature since the developing bone tissue depends on blood vessels to supply it with nutrients and oxygen [17,18].

Elastin is a protein whose main component is tropoelastin, a biologically active molecule that not only mediates cellular processes such as cytoskeletal organization, chemotaxis, proliferation, and differentiation, but also modulates the local tissue environment through the regulation of matrix proteases. Its mechanical stability, elasticity, and bioactivity make elastin a highly desirable candidate for the fabrication of biomaterials that can be used in wound healing and bone repair. Elastin is present in a number of native tissues and possesses biomimetic, physical, and biological characteristics that permit the incorporation of target cells and signaling molecules for extracellular matrix remodeling [19,20].

Chitosan is suitable for biomedical application in wound healing due to its high biocompatibility, biodegradability, porous structure, suitability for internal cell growth, osteoconduction, and antibacterial property. Degradable polymeric implants eliminate the need for a second surgery to remove the membrane [21]. As a graft material, chitosan can be used alone or in combination with other materials in the form of nanofibers for the delivery of drugs or bone growth factors [22,23].

Regenerative medicine aims to replace or repair organs through the administration of cells with regenerative and immunomodulatory properties. The use of biomaterials consisting of extracellular protein polymers is therefore advantageous because they have inherently desirable features for tissue regeneration, such as supporting cell activity and biodegradability [24,25]. Within this context, to obtain data on the effectiveness and mechanisms of these membranes and to identify perspectives for their clinical use in tissue engineering, the aim of this study was to analyze the osteoregenerative potential of natural collagen, elastin, and chitosan polymers applied to nasal bone defects of rats submitted or not to experimental alcoholism.

## 2. Materials and Methods

### 2.1. Animal Model

This study used 40 male Wistar rats (*Rattus norvegicus*), 12 weeks old and with an average weight of 350 g, provided by the Institute of Biomedical Sciences, University of São Paulo, and maintained in the bioterium of the Faculty of Medicine of Jundiaí, São Paulo. The animals were kept in suitable environments under a 12 h light/dark cycle at a controlled temperature (23 ± 1 °C) and received balanced ration for laboratory animals (Labina, Purina™, São Paulo, Brazil). A maximum of 4 animals were kept per box and, after surgery, they were individually allocated. The study was conducted according to the guidelines of the Declaration of Helsinki, and approved by the Institutional Ethics Committee on Animal Experimentation of the Faculty of Medicine of Jundiaí, Brazil (CEUA/FMJ), protocol code CEUA/FMJ No. 499/2012.

Furthermore, this experimental study was carried out according to the ARRIVE guidelines and based on the principles of NC3Rs. Throughout the experimentation, the animals were monitored for the expression of pain by observing whether the animal was apathetic, depressed, aggressive, or overexcited, such traits being variable in their usual behavior. Changes in walking, posture, or facial expression were also observed and the appearance, water consumption, food, and clinical symptoms were investigated. There were no complications that needed to be reported, and there was no disease or sign that strongly motivated the removal of an animal (clinical outcome) [26]

### 2.2. Induction of Alcoholism and Experimental Groups

Twenty of the forty rats were not exposed to alcohol and the other twenty were submitted to chronic alcohol ingestion. The animals were randomly distributed in the groups, without predetermined inclusion or exclusion criteria. The abstainer animals received only water ad libitum for 5 months. The alcoholic rats received increasing ethanol doses (5%, 10%, 15%, and 20%) diluted in water, with each dose being administered for one week. After this adaptation period, the animals received ethanol at 25% and, after that, the period of alcohol consumption of 4 months began, totaling 5 months of alcohol intake.

**Figure 1:**
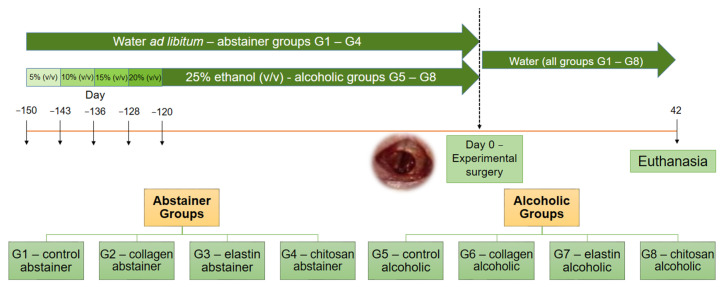
Study design timeline: 40 rats were divided into eight groups of 5 animals each. Twenty animals received tap water (abstainer groups G1 to G4) and twenty animals were submitted to experimental alcoholism with 25% ethanol (alcoholic groups G5 to G8), with previous gradual adaptation to increasing alcohol concentrations (−150 to −120 days). After 1 month of adaptation to experimental alcoholism, the concentration of 25% was maintained for an additional 4 months in groups G5 to G8. On day 0, all animals of the eight groups underwent surgery for the creation of an experimental nasal bone defect. The bone defect was filled with clot in G1 and G5, with collagen membrane in G2 and G6, with elastin membrane in G3 and G7, and with chitosan membrane in G4 and G8. After surgery, all animals continued to receive tap water and were euthanized at 42 days post-surgery.

All animals were submitted to surgery for the creation of an experimental nasal bone defect and were then divided into eight groups (*n* = 5/group) according to type of material used to fill the defect: collagen (Co), elastin (El), chitosan (Ch), or clot (control group, C). The 20 abstainer animals (Ab) were divided into four groups: G1—control/abstainer (C/Ab), G2—collagen/abstainer (Co/Ab), G3—elastin/abstainer (El/Ab), and G4—chitosan/abstainer (Ch/Ab). The 20 alcoholic animals (Al) were divided into the following four groups: G5—control/alcoholic (C/Al), G6—collagen/alcoholic (Co/Al), G7—elastin/alcoholic (El/Al), and G8—chitosan/alcoholic (Ch/Al) (Figure 1).

### 2.3. Fabrication of the Biomaterials

The collagen matrix was derived from bovine intestinal serosa and the elastin matrix from bovine auricular cartilage. The manufacturing process of matrices and their characterizations have been described in detail in previously published studies [16,27,28,29,30,31,32]. In summary, the bovine serosa and auricular cartilage were washed exhaustively in 0.9% saline (NaCl) and distilled water. The serosa and cartilage were hydrolyzed in an alkaline solution containing salts (sulfates and chlorides) and alkali metal and alkaline earth hydroxides. For hydrolysis, the bovine serosa was incubated for 24 h at a temperature not exceeding 25 °C and the bovine auricular cartilage for 96 h at 37 °C.

After this period, the matrices were equilibrated in another solution containing Na^+^, K^+^, and Ca^2+^ sulfates and chlorides. Excess salts were removed by washing in a solution of 3% boric acid (Sigma-Aldrich^TM^, Darmstadt, Germany) and deionized water, followed by a solution of 0.3% EDTA (Sigma-Aldrich^TM^, Darmstadt, Germany) and deionized water. After hydrolysis, the matrices were equilibrated in 0.01 mol/L H_3_PO_4_ (Sigma-Aldrich^TM^, Darmstadt, Germany) solution for 24 h for swelling and then lyophilized.

The chitosan matrix was obtained from the pens of *Loligo* squid by demineralization, deproteinization, and deacetylation. The matrix was characterized by conductometric titration and capillary viscometry and showed a degree of acetylation of 9% and a molecular mass of 4.37 × 10^5^ g/mol.

The collagen, elastin, and chitosan membranes were cut into 3 mm circular samples and prepared by the Biochemistry and Biomaterials Group of the São Carlos Institute of Chemistry (University of São Paulo—USP, Brazil).

### 2.4. Surgical Procedure

The animals were anesthetized by intramuscular injection of xylazine (6 mg/kg) (Rompum™, Bayer, São Paulo, Brazil) and ketamine (70 mg/kg) (Dopalen™, Ceva, São Paulo, Brazil). For analgesia, 50 mg/mL of tramadol hydrochloride (5 mg/kg body weight, Cronidor™, Agener União, São Paulo, Brazil) was injected intramuscularly 5 min before the surgical procedure. The rats were positioned in ventral decubitus on the operating table and the region of the nasal, frontal, and maxillary bone was shaved, followed by antisepsis with 2% chlorhexidine digluconate (Riohex™ 2%, Rioquímica, São José do Rio Preto, Brazil).

**Figure 2:**
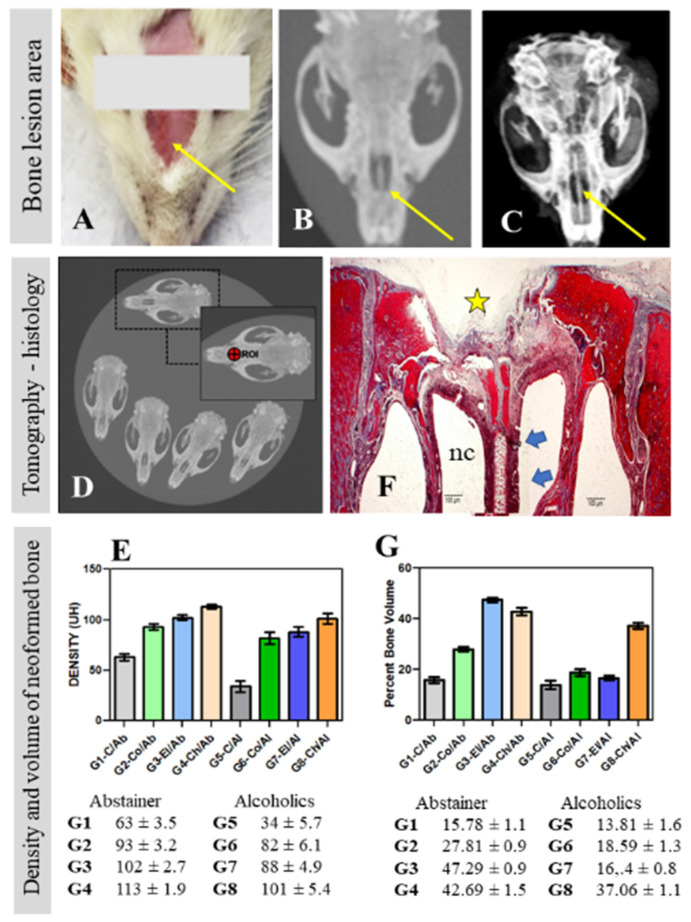
Surgical area (thin yellow arrows) in the nasal bone (**A**) for creation of the experimental bone defect (**B**,**C**). The samples were submitted to computed tomography (**D**) for calculation of bone density expressed as Hounsfield units (HU) in the bone region of interest (ROI) of the groups studied (**E**). Neoformed bone volume was calculated in the histological slides (**F**) of the bone defect area (yellow star) in all groups studied (**G**). Thick blue arrows indicate the nasal septum; nc = nasal cavity.

The nasal skin was cut sagittally and separated laterally for exposure of the periosteum and nasal bone. The periosteum was detached with the aid of a syndesmotome and a surgical drill coupled to an LB100 mini-motor (Beltec™, Araraquara, Brazil) was used to create a bone defect by removing a 3 mm circular bone fragment from the center of the nasal bone (Figure 2A–C). The bone perforation was sufficient to reach the inside of the nasal cavity, preserving the nasal septum. The defect was carefully cleaned with sterile gauze and physiological saline to remove secretions and possible bony fragments that could alter tissue regeneration.

Next, the defects were filled with one of the membranes (collagen matrix derived from bovine intestinal serosa in G2 and G6; elastin matrix derived from bovine auricular cartilage in G3 and G7; and chitosan matrix prepared from squid pens in G4 and G8), or were filled by the naturally formed clot (G1 and G5). After this step, the periosteum and skin were sutured with 6.0 silk suture (Ethicon™, Johnson & Johnson, São Paulo, Brazil). Each animal received one intramuscular dose of 0.1 mL/100 g body weight of veterinary antibiotic for small animals (Pentabiotico™, Zoetis, Campinas, Brazil), followed by rifampicin spray (Rifotrat™, Natulab, Rio do Sul, Brazil).

### 2.5. Euthanasia

All animals were euthanized within 6 weeks post-surgery by an intraperitoneal overdose (150 mg/kg body weight) of the anesthetic: xylazine–ketamine and thiopental (Thiopentax™, Cristália, Itapira, Brazil). After euthanasia of the animals, the skull was dissected and removed for macroscopic, radiographic, and tomographic evaluations. Next, specimens of interest were removed, fixed in 10% buffered formaldehyde, and processed for microscopic analysis.

### 2.6. Radiographic and Tomographic Analysis of the Surgical Area

The samples were submitted to radiography with an Yz-300mA Radiography Machine (Yangzhou Kangtai Medical Device Co™, Jiangsu, China), 100 mA focus, time of 0.06 s, 40 kV radiation and digitalized by the Agfa™ system (Agfa-Gavaert Corp., Mortsel, Belgium). Digital images were processed with the Agfa software for observation of the mineralization of the newly formed bone based on radiopaque and radiolucent features in the experimental area (Figure 2C).

The skulls of the rats were examined by multi-slice computed tomography with Toshiba Aquilion™ 16 and CT Scanner Gantry (CGGT-018A) systems (TSX-101A, Otawara, Tochigi, Japan). The grafted areas with possible osteosynthesis were selected and the bone region of interest (ROI) was delimited (width: 1.917 mm; height: 1.911 mm; depth: 6.0 mm = total area: 2.878 mm^2^) (Figure 2D).

The bone density was measured directly in this region, expressed as Hounsfield units (HU), which represent the relative density of body tissues on a calibrated grey level scale [33]. The HU values obtained were tabulated for the calculation of mean density and respective standard deviation (Figure 2E). The images were processed with the Osirix™ MD v.8.0.1 software (Pixmeo Sarl 2016, Geneva, Switzerland) for identification of the manipulated surgical areas grafted or not with the natural polymers.

### 2.7. Histological and Histomorphometric Analysis of the Surgical Area

The skulls containing the surgical area were fixed in 10% buffered formaldehyde, decalcified in hydrogen chloride, and reduced through frontal cuts with a Lupetec™ microtome (Lupe, São Carlos, Brazil) to obtain a block consisting of the nasal bone with the graft, nasal septum, and hard palate. The samples were transferred to cassettes for histological processing.

The samples were dehydrated and cleared in a Lupetec™ PT automatic tissue processor (Lupe, São Carlos, Brazil). After processing, the samples were vertically embedded in paraffin blocks and frozen at −5 °C. Semi-serial 5 µm cross-sections were obtained with a Lupetec™ MRP-03 rotary microtome, mounted on histological slides, and deparaffinized in a drying oven. The sections were stained with Masson’s trichrome (Figure 2F) for evaluation of the characteristics and volume of newly formed bone in the defect area (Figure 2G). Picrosirius Red was used to analyze the collagen fiber arrangement in the surgical area.

The images were obtained with a Motic™ BA310 light photomicroscope (Motic, Kowloon, Hong Kong) connected to a microcomputer equipped with the Motic Images Plus 2.0ML software (Motic China Group^TM^, Xiamen, China). The digitized images were saved in JPEG format, with 40× and 100× magnification. Bone neoformation in the grafted areas (Figure 2G) was quantified by means of histomorphometric analysis of the scanned images [34,35]. The total area analyzed corresponded to the total area of the surgical defect. This area was determined by identifying the borders of the nasal bone defect at the right and left margins of the surgical defect. These surfaces were connected by drawing lines following their curves. 

The bone area was delimited inside the total area. The latter was measured in μm^2^ and considered to be 100% of the area analyzed. The bone area was also measured in μm^2^ and calculated as the percentage of the total area. Two evaluators previously calibrated and blinded in relation to the groups and periods performed the constant analyses in the methodology.

### 2.8. Statistical Analysis

For the newly formed bone area, ANOVA and Tukey’s post-test were used to compare the results obtained between the groups, with *p* < 0.05 indicating statistical significance.

The number of animals in each treatment was estimated considering the minimum difference between the treatment means, standard deviation of the residue, test power, and significance level of 3.0, 1.4, 0.80 and 0.05, respectively. These values were used based on previous information to estimate the percentage volume of new bones formed.

Data were submitted to ANOVA and subsequent Tukey test at 5% probability. The ANOVA assumptions, normality, and homogeneity of the variances were verified by the Shapiro–Wilk and Bartlett tests, both at 5% probability. All analyses were performed using BioEstat 5.3 software™ (Mamirauá Institute, Manaus, Brazil).

## 3. Results

### 3.1. Radiographic and Tomographic Analysis

**Figure 3:**
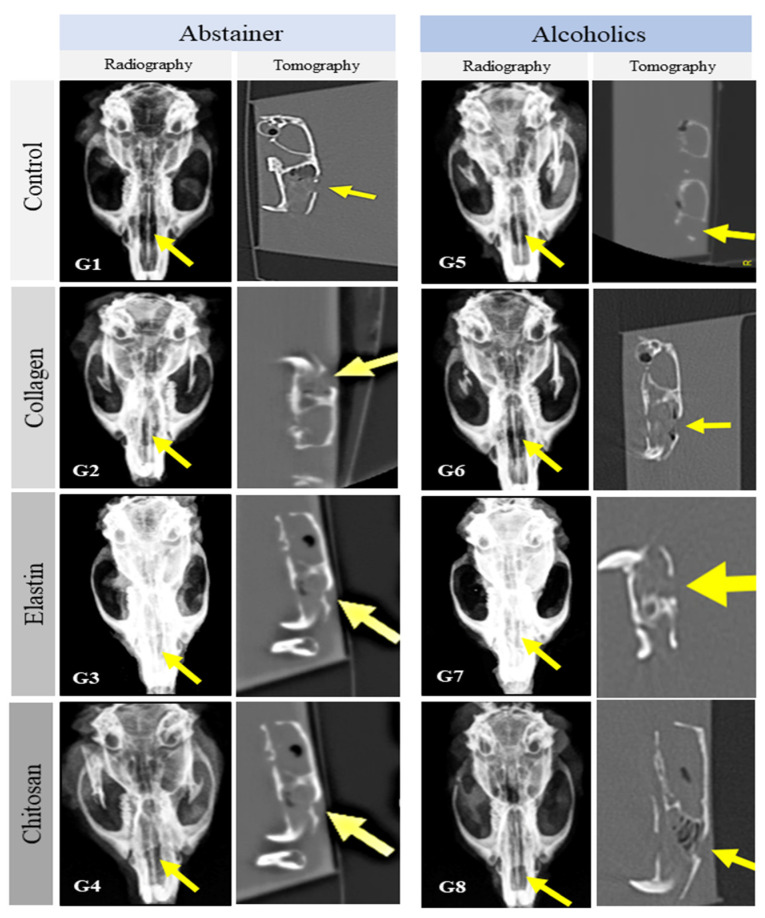
Radiographic and tomographic findings in the surgical area. The bone defect (yellow arrows) was not completely closed in any of the studied groups, and no bone deformity was observed. Abstainer animals (Ab): G1—control/abstainer (C/Ab), G2—collagen/abstainer (Co/Ab), G3—elastin/abstainer (El/Ab), and G4—chitosan/abstainer (Ch/Ab). Alcoholic animals (Al): G5—control/alcoholic (C/Al), G6—collagen/alcoholic (Co/Al), G7—elastin/alcoholic (El/Al), and G8—chitosan/alcoholic (Ch/Al).

No radiological signs indicative of pathological alterations in the surgical area or adjacent regions were observed in any of the groups studied who received grafts, suggesting the compatibility of the matrices and the maintenance of nasal bone architecture. There were no deformities, bone resorption, cyst formation, avascular necrosis, or secondary fractures. A radiolucent image indicating the proliferation of connective tissue in the surgical area predominated in G1 (abstainer control, C/Ab) and G5 (alcoholic control, C/Al). However, a discrete radiopaque image resulting from the presence of the natural polymer matrices was observed in G2 to G4 (abstainer) and G6 to G8 (alcoholic) (Figure 3).

The mean densities in HU obtained by tomographic examination of the defect areas were 63 ± 3.5, 93 ± 3.2, 102 ± 2.7, 113 ± 1.9, 34 ± 5.7, 82 ± 6.1, 88 ± 4.9, and 101 ± 5.4 in G1 to G8, respectively (Figure 2E).

### 3.2. Histological Analysis of the Surgical Area

**Figure 4:**
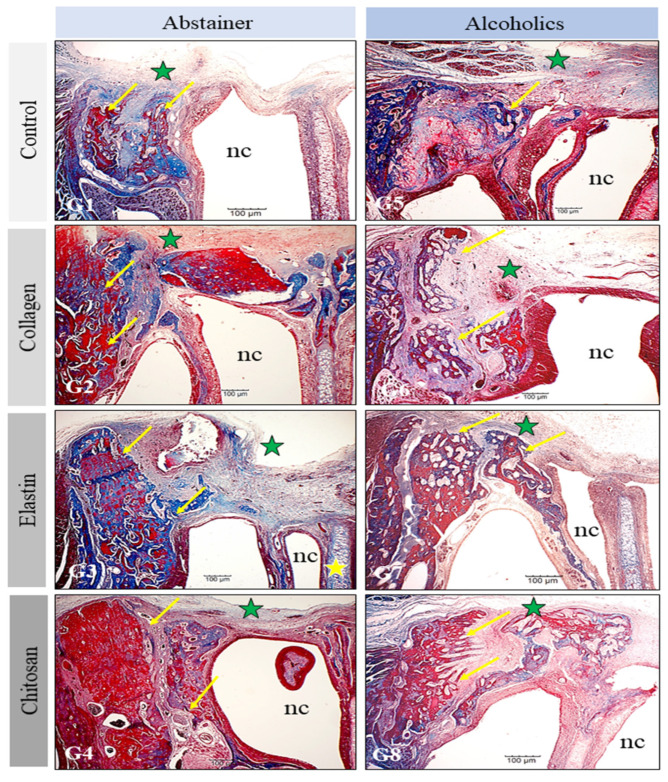
Histological findings in the surgical areas (green star) of the groups studied (G1–G8). The newly formed bone (arrows) originates from the margins of the defect and is more compact in the abstainer groups and more trabecular in the alcoholic groups. The nasal cavity (nc) and nasal septum (yellow star) are preserved in all groups. Bar: 100 µm.

In all groups analyzed, the space and structures of the nasal cavity, such as the nasal turbinates and septum, were intact. There were no bone deformities or signs indicative of an acute or chronic inflammatory process. Bone neoformation occurred from the margins of the original bone but was not sufficient to permit total bone repair during the experimental period standardized in this study. In the abstainer groups, the newly formed bone was more voluminous and compact, while bone with a more trabecular, dispersed, and porous appearance predominated in the alcoholic groups (Figure 4).

In G1 (C/Ab) and G5 (C/Al), who did not receive the membrane grafts, the formation of thin and linear connective tissue was observed, which covered the whole extent of the bone defect. The formation of new bone occurred near the margins of the native bone and was more marked in G1 (C/Ab) compared to G5 (C/Al). In G2 (Co/Ab) and G6 (Co/Al), whose nasal bone defects were filled with the collagen membranes, the formation of new bone occurred both at the margins of the defect and in areas without contact with the native bone, particularly in G2, in which bone neoformation was also identified on the nasal septum.

In G6, connective tissue was observed at different sites and amidst foci of more porous bone neoformation. In addition, the margins of the defect in G6 contained less newly formed bone than in G2. In G3 (El/Ab) and G7 (El/Al), whose nasal bone defects were filled with the elastin membranes, the formation of thick bone originating from the margins of the defect was observed in G3. In G7, dispersed areas of neoformation of porous and immature bone were identified, which were surrounded by connective tissue (Figure 4). In G4 (Ch/Ab) and G8 (Ch/Al), whose nasal bone defects were filled with the chitosan membranes, the neoformation of denser bone was observed in G4, while the bone was more immature and dispersed in G8, with an interposition of connective tissue between the foci of bone formation (Figure 4).

Based on the morphological images of Picrosirius Red, three levels of birefringence brightness intensity were revealed (red, green, and yellow), indicating a spatial organization with a slight difference in heterogeneity between the experimental groups. However, the images of abstainer groups (G1–G4) allowed us to observe that they were more intense in relation to alcoholics (G5–G8).

**Figure 5:**
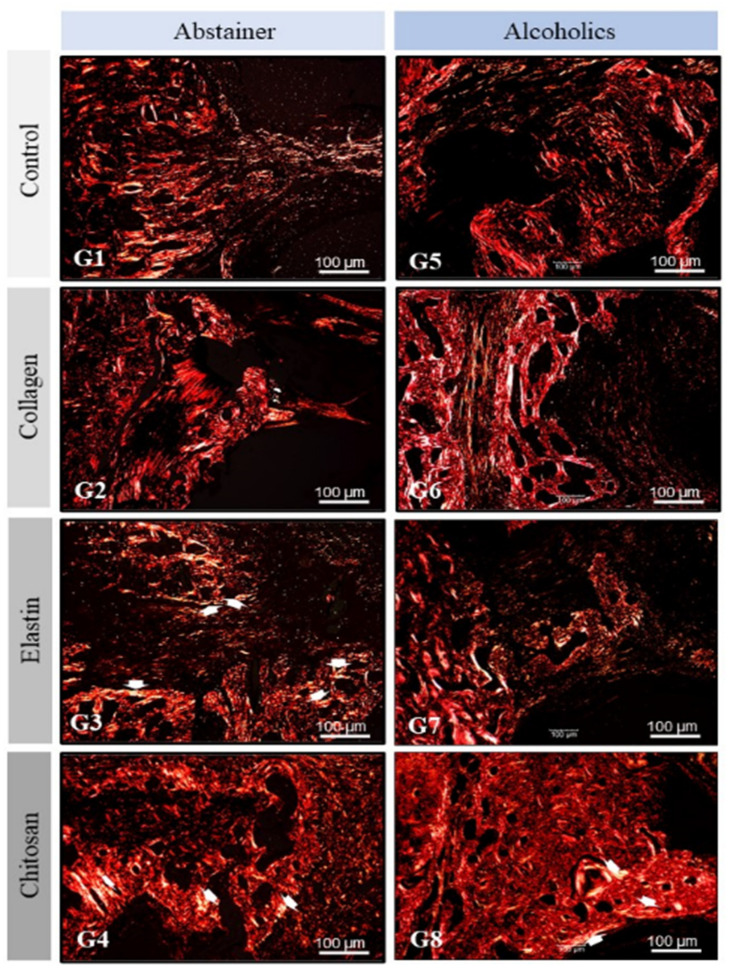
Polarized light analysis of the extracellular matrix in the surgical areas of the groups studied (G1–G8). The collagen fibers exhibited good birefringence in all groups but were disorganized in the alcoholic groups (G5–G8), with yellow birefringence transacting to green (white arrow). Bar: 100 µm.

In G3 (elastin/abstainer—El/Ab), G4 (chitosan/abstainer—Ch/Ab), and G8 (chitosan/alcoholic—Ch/Al), we found crossing points of thick and thin collagen fibers with yellow birefringence transacting to the green, forming circular structures, which shows the maturation phase of the organic matrix. The fibrils extended from the marginal bone level to isolated loci in the center of the defect (see white arrow), creating an orderly compaction of collagen bundles, which exhibit wavy, three-dimensional structures (Figure 5).

### 3.3. Histomorphometric and Statistical Analysis

The percent volume of newly formed bone in all groups (mean ± SD, standard deviation) in the defect areas quantified by histomorphometric analysis is shown in Table 1 and Figure 2G. There was no significant difference between groups G1, G5, and G7 or between G6 and G7.

Comparison of each abstainer group with its respective alcoholic group, i.e., G1 vs. G5, G2 vs. G6, G3 vs. G7, and G4 vs. G8, showed a reduction in the volume of newly formed bone of 12.5%, 33.2%, 65.1%, and 13.2%, respectively, demonstrating the negative effects of alcoholism on bone tissue even when biomaterials are used for stimulation of the bone repair process.

## 4. Discussion

Maxillofacial fractures are associated with a high rate of morbidity, and can have severe consequences such as bone deformities and significantly reduced upper airways. Some of these injuries may heal naturally because of the regenerative capacity of bone tissue; however, in cases of marked bone loss, bone grafts or biomaterials that mimic the functions and components of the bone matrix are necessary.

Tissue engineering has demonstrated great potential for the creation of biomaterials that are capable of developing into functional tissues [11]. Biomaterials are traditionally classified according to their origin into synthetic or natural materials. Natural biomaterials for use as membranes can be autologous, such as platelet-rich fibrin [36], fibrin sealants [37,38,39,40,41], or xenogenous [42,43,44]. The three membranes used in the present study belong to the latter category. Correlating the histological and morphological characteristics of the tissue repair process, the results of this study showed that the xenogenous collagen, elastin, and chitosan membranes used were biocompatible and promoted the proliferation of osteogenic cells at the site of injury in the nasal bone of rats [23,30].

Notably, the quality and preservation of the tissues in the surgical area and adjacent to it observed upon macroscopic inspection, in the absence of clinical signs of infectious processes and/or necrosis in soft or bone tissue, indicate the biocompatibility of the biomaterials [32,45]. This fact was observed in all groups receiving the extracellular matrix implants (G2 to G4 and G6 to G8). In addition, radiographic examination revealed no images of bone rarefaction, indicating absorption due to infectious processes but only demonstrated the quality of the image, radiolucency or radiopacity, compatible with the process of bone regeneration [46].

Although alcoholism did not affect the biocompatibility of the membranes, the evaluation of bone mineral density by computed tomography revealed its harmful effects. In this respect, rats of the alcoholic groups (G5–G8) exhibited lower bone mineral density in the defect area (34 ± 5.2, 82 ± 6.1, 88 ± 4.9, and 101 ± 5.4, respectively) than the abstainer groups (G1-G4) (63 ± 3.5, 93 ± 3.2, 102 ± 2.7, and 113 ± 1.9, respectively). Alcohol intake decreases osteoblastic activity with an increase in osteoclastic activity, leading to a decrease in bone mineral density and, with prolonged abuse, osteoporosis [47,48].

Histological and histomorphometric analysis also demonstrated lower bone neoformation, with smaller bone areas, in all groups of alcoholic rats when compared to the respective abstainer group: G1 vs. G5 (15.78 ± 1.19 vs. 13.81 ± 1.60), G2 vs. G6 (27.81 ± 0.91 vs. 18.59 ± 1.37), G3 vs. G7 (47.29 ± 0.97 vs. 16.54 ± 0.89), and G4 vs. G8 (42.69 ± 1.52 vs. 37.06 ± 1.17). However, there were no significant differences between the control groups (G1 and G5), probably because the absence of a membrane resulted in extremely low levels of newly formed bone in both groups. The insertion of grafts, mainly in the form of membranes, can hinder the invasion of soft tissues adjacent to the defect, allowing a reinforcement structure that favors the growth of bone cells [14,49,50].

Furthermore, the significant difference between the grafted groups of alcoholic and abstainer rats might be explained by the harmful effect of alcohol exposure on bone tissue repair, in which alcohol causes derangement of the inflammatory response, with consequent alterations in the production of cytokines and chemokines by different types of cells. These events, in turn, influence the responses of other types of cells, altering the basic Wnt signaling processes, which are essential for wound repair [8].

Among the biomaterials used in this study, those used in G2 (27.81 ± 0.91), G6 (18.59 ± 1.37), and G7 (16.54 ± 0.89) showed the poorest performance. The main disadvantage of collagen-based membranes, used in G2 and G6, is their rapid degradation due to the enzymatic activity of macrophages and polymorphonuclear leukocytes, in which differences in the degradation pattern of the membranes can have clinical implications [51]. In the present study, the collagen matrices did not seem to offer support for efficient bone growth, as observed 6 weeks after the grafting procedure. Some studies suggest that the physicochemical conditions (degradation) of collagen implants can be improved by treatment with tetracycline [52,53] or glutaraldehyde [50] and that mechanical resistance can be added by reinforcing collagen with elastin-like polypeptides [54] or by coating it with apatite [55].

Regarding the groups grafted with elastin, rats not exposed to alcoholism (G3) exhibited the highest volume of newly formed bone in this study (47.29 ± 0.97). However, when submitted to alcoholism (G7), the animals had the worst result among all groups studied (16.54 ± 0.89). Further studies are needed to elucidate the reason for this large difference in the results between alcoholics and abstainers when an elastin membrane is used. One hypothesis is that the subproducts of alcoholism interfere with cell–cell interactions during degradation of the elastin membrane [56]. Alcohol consumption, depending on the time and amount of abuse, may be correlated with upregulation of the enzymatic activity of matrix metalloproteinase-2 (MMP-2), which is coincident with altering the composition of the extracellular matrix through the degradation of the components of elastin, mainly in the vascular system [57].

Secretome also features prominently in regenerative medicine, in addition to supporting the extracellular matrix and biomaterials in tissue replacement. The developmentally immature profile of fetal human amniotic fluid stem cells (hAFS) may be recapitulated by their secretome formulations endowed with a more pronounced pro-vasculogenic, pro-regenerative, and rejuvenating secretome. Perinatal hAFS still retain a relevant paracrine profile via the expression of factors related to endothelial cell migration, immune-modulatory, anti-inflammatory, and neurotrophic potential similar to fetal hAFS [58]. Regenerative medicine consists of cell transplantation, tissue engineering, drug research and gene therapy, with “high-activity cell utilization” being an essential factor. Furthermore, the interaction of biomaterials and immune cells close to target cells is also an important factor because this interaction leads to the immune response. The development of regenerative medicine based on biomaterials is directly related to the reaction of the cells of the immune system [59].

Moreover, secretome is not only a topic depending on the cell releasing of topical factors, but also on other ectopic co-factors that could interact/alter tissue adaptation after a repeated micro-trauma. Reactive oxygen species (ROS) are directly related to the recruitment of neutrophils, a central factor of the inflammatory process and critical for the elimination of pathogens. The number of neutrophils at sites of infection must be carefully regulated to ensure that enough neutrophils are recruited for efficient clearance, minimizing over-recruitment that drives immune pathology [60].

An ideal structure for bone application that contributes to tissue engineering and organ repair must be extremely porous, biocompatible, and biointeractive, with individualized resorption and adequate mechanical properties. The association of one or more bioactive or biointeractive fillers represents an interesting strategy in bone tissue engineering. Regenerative dentistry seeks a scaffold capable of ensuring the regenerative healing of the periapical and alveolar bone, allowing for the preservation of teeth. Innovative clinical approaches combining highly porous and biointeractive scaffolds colonized with autologous stem cells from periapical cysts represent a promising strategy for the regenerative healing of periapical and alveolar bone [61].

Regardless of liquid diet (abstainer or alcoholics), animals that received chitosan grafts had the best quantitative results of bone density (expressed in Hounsfield units—HU) in the bone region of interest, with 113 ± 1.9 in abstainer (G4—Ch/Ab) and 101 ± 5.4 in alcoholics (Ch/Al) (Figure 2E). In relation to the neoformed bone volume, evaluated histologically in the bone defect area, chitosan also obtained good results, being the one with the highest formation in alcoholic animals (G8—Ch/Al; 37.06 ± 1.1) and the second highest in the bone formation of abstainers (G4—Ch/Ab; 42.69 ± 1.5) (Figure 2G). A hypothesis to explain these results is the greater resistance of the chitosan matrix to solubility and degradation [21,62,63].

Analysis of the Picrosirius Red-stained histological sections showed the expressive formation of fibrous tissue in the groups of rats submitted to alcoholism, with the observation of thicker, crossed fibers without apparent orientation in the areas adjacent to the grafts and of fibers interposed among foci of ossification. These findings were more evident in G5, G6, and G7 and indicate the interference of alcoholism with the mechanisms of bone formation and/or degradation [6]. The inhibition of osteoblast differentiation and proliferation pathways, caused by chronic alcohol intake, contribute to the formation of fibrous tissue [64].

Despite all the analyses performed in this study, not performing an immunohistochemical analysis could be considered a limitation. The inaccessibility to research centers during the period of the new coronavirus pandemic, since March 2020, and the high cost of antibodies led to this limitation.

## 5. Conclusions

This study aimed to evaluate the use of collagen, elastin, or chitosan biomaterial for bone reconstruction in rats submitted or not to experimental alcoholism. The extracellular matrices of collagen, elastin, and chitosan implanted in nasal bone defects of rats exhibited biocompatibility. Osteogenesis and bone density were more expressive after the application of the elastin matrix in abstainer rats and the chitosan matrix in abstainers and alcoholics. Chronic alcohol intake predisposes to a greater formation of fibrous connective tissue, slowing down the bone regeneration process, even using natural polymeric biomaterials as scaffolds.

There is a need for further studies, with the current trend towards an increase in the number of cases of extensive bone loss that do not repair themselves and that require orthopedic and dental procedures in patients who chronically use alcoholic beverages. The association of grafting materials with therapies that reduce recovery time and help bone neoformation, such as photobiomodulation [65,66], create perspectives for studies to be carried out in the future.

## Figures and Tables

**Table 1:** Percent volume of newly formed bone.

Groups	Mean ± SD
G1—control/abstainer (C/Ab)	15.78 ± 1.19 fg
G2—collagen/abstainer (Co/Ab)	27.81 ± 0.91 d
G3—elastin/abstainer (El/Ab)	47.29 ± 0.97 a
G4—chitosan/abstainer (Ch/Ab)	42.69 ± 1.52 b
G5—control/alcoholic (C/Al)	13.81 ± 1.60 g
G6—collagen/alcoholic (Co/Al)	18.59 ± 1.37 e
G7—elastin/alcoholic (El/Al)	16.54 ± 0.89 ef
G8—chitosan/alcoholic (Ch/Al)	37.06 ± 1.17 c

Analysis was performed between the different groups (G1–G8): ANOVA followed by the Tukey’s test. Mean ± standard deviation, SD (*n* = 5 animals/group), where different lowercase letters indicate statistically significant differences (a ≠ b ≠ c ≠ d ≠ e ≠ f ≠ g; *p* < 0.05).

## Data Availability

The data presented in this study are available on request from the corresponding author. The data are not publicly available due to being a part of a master’s dissertation not yet deposited in a public repository.

## Sample Availability

Samples of the chitosan, elastin, and collagen matrices are available from the authors.
